# Knowledge and attitudes toward human papillomavirus and its vaccine among medical students

**DOI:** 10.1590/1806-9282.20260171

**Published:** 2026-07-10

**Authors:** Nur Erdem, Betül Battaloğlu İnanç

**Affiliations:** 1 Bahçelievler District Health Directorate, Department of Public Health – Istanbul, Türkiye.; 2 Muğla Sıtkı Koçman University, Faculty of Medicine, Department of Family Medicine – Muğla, Türkiye.

**Keywords:** Human papillomavirus, HPV vaccine, Medical students, Awareness, Knowledge

## Abstract

**OBJECTIVE:**

The aim of this study was to evaluate knowledge and attitudes regarding human papillomavirus infection and vaccination among medical students and to examine differences according to gender and academic year.

**METHODS:**

This cross-sectional study was conducted during the 2024–2025 academic year among medical students enrolled at Muğla Sıtkı Koçman University Faculty of Medicine. Data were collected through a voluntary online questionnaire. The human papillomavirus Knowledge Scale (33 items; theoretical range 0–33) was used to assess knowledge. Results are presented as median (minimum–maximum). Group comparisons were performed using non-parametric tests, and multiple linear regression analysis was conducted to identify independent predictors of knowledge.

**RESULTS:**

A total of 372 students participated (56.5% female; mean age 21.5 years). The median total human papillomavirus knowledge score was 20.5 (0–31). Female students demonstrated significantly higher knowledge scores than male students (p<0.001). Knowledge increased significantly with the advancing academic year (p<0.001), with first- and second-year students exhibiting the lowest scores across all subscales. Multiple linear regression analysis revealed that academic year and prior awareness of human papillomavirus testing were the strongest independent predictors of total knowledge (R^2^=0.411, p<0.001), followed by female gender, academic year, and alcohol use.

**CONCLUSION:**

Although the overall level of human papillomavirus awareness was found to be satisfactory, knowledge levels were lower among male students, those in the early years of medical education, and individuals with unfavorable health behaviors (such as alcohol use). Providing comprehensive education from the early years of training may enhance future physicians’ capacity to deliver effective preventive counseling.

## INTRODUCTION

Human papillomavirus (HPV) is one of the most common sexually transmitted infections worldwide and represents a significant public health concern affecting both women and men. The World Health Organization reports that more than 350 million new sexually transmitted infection cases occur annually among individuals aged 15–49 years, with HPV being a major contributor to infection-related morbidity and cancer burden^1^. While HPV infection is often asymptomatic and may resolve spontaneously, persistent infection with high-risk HPV types can lead to serious health outcomes, including anogenital cancers (cervical, vulvar, vaginal, anal, and penile cancers) as well as head and neck cancers^2,3^. HPV types 16 and 18 are responsible for approximately 70% of cervical cancer cases worldwide, making cervical cancer one of the leading malignancies among women of reproductive age^2–4^. Although condom use reduces the risk of HPV transmission, it does not provide complete protection. Therefore, cervical cancer prevention primarily relies on effective screening programs for women and trans men with a cervix and HPV vaccination strategies^5–8^. Numerous studies have demonstrated that HPV vaccination significantly reduces the incidence of HPV-related infections, precancerous lesions, and associated malignancies^3,5,9^.

In Turkey, HPV vaccination has been available since 2007, initially with the quadrivalent vaccine targeting HPV types 6, 11, 16, and 18, followed by the introduction of the bivalent vaccine in 2008. Since 2023, the nonvalent HPV vaccine, which covers additional oncogenic types (31, 33, 45, 52, and 58), has been available^5,9^. The World Health Organization recommends routine HPV vaccination for children aged 9–14 years prior to the onset of sexual activity^5^. In addition, vaccination of males has been shown to contribute to the prevention of genital warts, anal cancer, and oropharyngeal cancer, while also enhancing herd immunity and reducing HPV transmission among women^2,3,7,10^.

Healthcare professionals play a critical role in the success of HPV prevention strategies, as their knowledge, attitudes, and recommendations strongly influence public acceptance of screening and vaccination programs^6^. Previous studies conducted among medical students and healthcare trainees have shown that higher levels of HPV-related knowledge are associated with more positive attitudes toward vaccination and stronger intentions to recommend the vaccine in future clinical practice^7,8^. In Turkey, studies evaluating HPV knowledge, attitudes, and behaviors have largely focused on physicians, intern doctors, and nursing students, with relatively limited data available regarding medical students across different years of education^5^. Moreover, regional data reflecting variations in medical education and clinical exposure remain scarce. Assessing the knowledge and awareness levels of medical students is particularly important, as these individuals will soon assume active roles in patient counseling, preventive medicine, and public health advocacy.

Therefore, this study aimed to evaluate the knowledge levels and attitudes of medical students regarding HPV infection and vaccination, to identify differences according to gender and academic year, and to contribute evidence that may support the development of targeted educational strategies. To the best of our knowledge, this is the first study conducted in Muğla province addressing this topic among medical faculty students.

## METHODS

### Data collection

This cross-sectional study was conducted among medical students enrolled at Muğla Sıtkı Koçman University Faculty of Medicine during the 2024–2025 academic year (n=1,353). The required minimum sample size was calculated as 300 participants using G*Power 3.1.9.7, assuming a 95% confidence level and a 5% margin of error. The study was carried out with the voluntary participation of 372 students: 62 first-year, 60 second-year, 64 third-year, 62 fourth-year, 61 fifth-year, and 63 sixth-year students. The final sample size exceeded the minimum required number, thereby increasing statistical precision.

Researchers visited lecture halls, informed students about the purpose and scope of the study, and invited them to participate. Participants were recruited using a convenience sampling method based on voluntary participation. The survey link was distributed through official communication channels and student networks. As participation was voluntary, selection bias may have occurred, particularly if students with a greater interest in HPV-related topics were more likely to participate. Participation was based entirely on voluntary consent, and an informed consent form was included on the first page of the questionnaire.

Participants who agreed to take part completed a sociodemographic form consisting of 13 questions (gender, class year, age, socioeconomic status, parental education level, smoking–alcohol habits, presence of chronic disease, and regular medication use) and the HPV Knowledge Scale to determine their level of knowledge about HPV and its vaccine. The “Human Papillomavirus Knowledge Scale,” developed by Waller et al. in 2013 and known in the literature as the “HPV Knowledge Scale,”^11^ was adapted to Turkish with validity and reliability established by Demir Bozkurt in 2023 and applied in this study^12^. The scale consists of 33 items. Scores were evaluated both for the total scale and for subdimensions. The HPV Knowledge Scale consists of 33 dichotomously scored items (correct=1, incorrect=0), yielding a theoretical score range of 0–33, with higher scores indicating greater knowledge. The HPV Knowledge Scale consists of four subdimensions: Subscale 1: General knowledge about HPV (Items 1–16), Subscale 2: knowledge about the HPV test (Items 17–22), Subscale 3: General knowledge about the HPV vaccine (Items 23–27), and Subscale 4: Knowledge about the vaccination program (Items 28–33). The Cronbach’s alpha coefficient calculated to assess the reliability of the scale was 0.90.

### Inclusion criteria

Being a student at Muğla Sıtkı Koçman University Faculty of Medicine during the 2024–2025 academic year, agreeing to participate in the survey and complete the questionnaire fully.

### Ethical approval

This study was reviewed and approved by the Medical Sciences Ethics Committee of Muğla Sıtkı Koçman University Faculty of Medicine with decision number 149 dated 07/11/2024 and protocol number 240170. The study was conducted in accordance with the Declaration of Helsinki and in compliance with the ethical standards of our country.

### Statistical analysis

Collected data were analyzed using SPSS (Statistical Package for the Social Sciences) for Windows, version 27.0. Descriptive statistics were presented as median (minimum–maximum) for continuous variables and as frequency (n) and percentage (%) for categorical variables. Normality of continuous variables was assessed using the Kolmogorov-Smirnov test. As total and subscale scores were not normally distributed, non-parametric tests were applied. The Kruskal-Wallis test was used for comparisons among more than two groups, followed by post hoc Mann-Whitney U tests with Bonferroni-adjusted significance levels where appropriate. For two-group comparisons, the Mann-Whitney U test was applied. Categorical variables were analyzed using the chi-square test. Multiple linear regression analysis was conducted to identify independent predictors of knowledge scores. To control for Type I error inflation due to multiple comparisons, Bonferroni correction was applied (adjusted significance threshold p<0.01). A p<0.05 was considered statistically significant unless otherwise specified.

## RESULTS

A total of 372 medical students participated in the study; 56.5% were female, and the mean age was 21.5 years. Female students demonstrated significantly higher knowledge scores than males (p<0.001) and reported greater awareness of HPV and HPV vaccination. The median total HPV knowledge score was 20.5 (0–31). Knowledge levels increased significantly with the advancing academic year (p<0.001) (Table 1). First- and second-year students had consistently lower scores across all subscales and the total scale compared with upper-class students. Differences between class years were particularly pronounced in Subscale 3 (HPV vaccine knowledge) and Subscale 4 (vaccination program knowledge). A significant difference in total scores was also observed between third-year preclinical students and sixth-year students (p<0.001) (Table 2). Awareness of HPV testing (p=0.008) and HPV vaccination (p=0.002) similarly increased with academic progression (Tables 2 and 3). Students who had previously heard of the HPV test achieved significantly higher scores across all subscales and on the total scale compared with those who had not heard of it or responded “I don’t know” (p<0.001) (Table 2).

**Table 1:** Human papillomavirus knowledge scale observed scores according to class year.

Scale/subscale	1st year	2nd year	3rd year	4th year	5th year	6th year	Total	Test statistic	p-value
Subscale 1 (general HPV knowledge)	10 (0–15)	10.5 (0–14)	12 (3–15)	13 (8–16)	13 (6–16)	14 (0–16)	11.6 (0–16)	*χ* ^2^=9.00	<0.001*
Subscale 2 (HPV test knowledge)	2 (0–6)	2 (0–6)	3 (0–6)	3 (0–6)	4 (0–5)	4 (0–6)	2.99 (0–6)	*χ* ^2^=83.04	<0.001*
Subscale 3 (HPV vaccine knowledge)	3 (0–5)	4 (0–5)	4 (0–5)	4 (1–5)	4 (0–5)	4 (0–5)	3.51 (0–5)	*χ* ^2^=32.09	<0.001*
Subscale 4 (vaccination program knowledge)	1 (0–4)	1 (0–5)	2 (0–5)	3 (0–5)	3 (0–6)	3 (0–6)	2.37 (0–6)	*χ* ^2^=91.11	<0.001*
HPV knowledge scale (total)	16.5 (0–26)	17 (0–28)	22 (3–28)	23 (13–29)	24 (11–30)	25 (0–31)	20.5 (0–31)	*χ* ^2^=119.90	<0.001*

Values are presented as median (minimum–maximum).

* Kruskal-Wallis test was used. HPV: human papillomavirus.

**Table 2:** Distribution of human papillomavirus knowledge scale observed scores according to categorical variables.

Variable	Category	Subscale 1	Subscale 2	Subscale 3	Subscale 4	Total score	Statistical test
Gender	Female (n=210)	12 (1–16)	3 (0–6)	4 (0–5)	3 (0–6)	23 (4–31)	Z=−4.025; p<0.001**
	Male (n=162)	12 (0–16)	3 (0–6)	4 (0–5)	2 (0–5)	20 (0–30)	
Alcohol use	Yes (n=176)	12 (0–16)	3 (0–6)	4 (0–5)	2 (0–5)	20 (0–30)	Z=−4.257; p<0.001**
	No (n=196)	13 (0–16)	3 (0–6)	4 (0–5)	3 (0–6)	23 (0–31)	
Chronic disease	Present (n=46)	13 (2–16)	3 (0–6)	4 (0–5)	3 (0–5)	24 (3–30)	Z=−2.283; p=0.022**
	Absent (n=326)	12 (0–16)	3 (0–6)	4 (0–5)	2 (0–6)	21.5 (0–31)	
Regular medication use	Present (n=67)	13 (2–16)	3 (0–6)	4 (0–5)	3 (0–6)	23 (3–31)	Z=−2.566; p=0.010**
	Absent (n=305)	12 (0–16)	3 (0–6)	4 (0–5)	2 (0–5)	21 (0–30)	
Awareness of HPV test	Yes (n=323)	12 (0–16)	3 (0–6)	4 (0–5)	3 (0–6)	22 (0–31)	*χ* ^2^=36.051; p<0.001*
	No (n=34)	11 (0–15)	2 (0–6)	3 (0–5)	1 (0–5)	16.5 (0–29)	
	I don’t know (n=15)	9 (0–14)	2 (0–4)	2 (0–5)	1 (0–3)	17 (0–21)	

Values are presented as median (minimum–maximum).

* Kruskal-Wallis test and

** Mann-Whitney U tests were used as appropriate. HPV: human papillomavirus.

**Table 3:** Awareness of human papillomavirus, human papillomavirus testing, human papillomavirus vaccination, chronic disease status, and smoking status among medical students.

Variable	Category	n (%)	p-value
Awareness of human papillomavirus	Yes	362 (97.3)	0.057
	No/I do not know	10 (2.7)	
Awareness of the HPV test	Yes	323 (86.8)	0.008***
	No/I do not know	49 (13.2)	
Awareness of the HPV vaccine	Yes	352 (94.6)	0.002***
	No/I do not know	20 (5.4)	
Chronic disease status	Present	46 (12.4)	0.002***
	Absent	326 (87.6)	
Smoking status	Smoker/Occasional/ Former	147 (39.5)	0.008***
	Never smoked	225 (60.5)	

*** Chi-square test. HPV: human papillomavirus.

Multiple linear regression analyses identified academic year and prior awareness of HPV testing as the strongest independent predictors across all subscales and the total knowledge score (all p<0.001). For the total score, the model explained 41.1% of the variance [R^2^=0.411; adjusted R^2^=0.401; F(6,365)=42.463, p<0.001]. Academic year (B=1.544, p<0.001) and prior HPV test awareness (B=−test awa<0.001) were the most influential predictors. Female gender was positively associated with knowledge (B=2.230, p<0.001), whereas alcohol consumption demonstrated a negative association (B=−2.028, p<0.001). Smoking status and chronic disease status were not significant predictors in the adjusted model. Spearman correlation analyses supported these findings. Academic year showed a strong positive correlation with total knowledge (r=0.543, p<0.001), while female gender was weakly to moderately associated (r=0.226, p<0.001). Alcohol use was negatively correlated with knowledge (r=−0.214, p<0.001). All subscales were strongly correlated with the total score (r=0.699–0.810, p<0.001), confirming internal consistency. After Bonferroni correction (p<0.01), academic year, gender, alcohol use, and HPV-related awareness variables remained statistically significant.

## DISCUSSION

HPV represents a major public health concern due to its established role in the development of multiple cancers in both women and men. As emphasized in the literature, increasing knowledge, awareness, and acceptance of HPV and its vaccination is critical for the effective prevention of HPV-related diseases^8,10^.

The present study evaluated HPV-related knowledge and awareness among medical students and demonstrated that awareness—operationalized as having heard of HPV and the HPV test—was significantly associated with female gender and advancing academic year. Consistent with studies conducted among university and medical students in Brazil, the United States, China, and Turkey, our findings showed that female students had significantly higher HPV knowledge scores than male students^3,5,13,14,15,16^. This difference may be explained by the perception of HPV as a “women’s disease,” leading female students to perceive themselves at direct risk and to seek health-related information more actively. Similar patterns have also been reported in systematic reviews and meta-analyses assessing HPV awareness and vaccination attitudes^8^.

Higher knowledge levels among female students may reflect not only personal and individual experiences but also long-standing cervical cancer prevention strategies that have primarily targeted girls and young women. Within this framework, lower HPV knowledge scores observed among male students^2,3,7,10^ may be interpreted in light of gender roles, limited awareness of HPV’s association with penile, anal, and oropharyngeal cancers, and the historically later emphasis on HPV vaccination for males^10,13^.

Furthermore, lower knowledge levels observed among first- and second-year students and among those reporting alcohol use suggest the presence of potential risk groups. These findings indicate that relying solely on cumulative academic exposure over time may be insufficient. Instead, structured and comprehensive HPV education should be introduced early in medical training.

As the academic year progressed, HPV knowledge and awareness increased. First- and second-year students scored significantly lower across all subscales of the HPV Knowledge Scale compared with senior students. These findings are consistent with previous studies conducted among medical students, interns, and nursing students^4,5,9,13,16,17^. While an increase in knowledge with an advancing academic level is expected, our study further demonstrated that knowledge was significantly associated not only with academic progression but also with prior awareness of HPV and the HPV test.

The marked increase in knowledge beginning in the third academic year may be attributed to the transition to clinical training and increased exposure to clinical sciences. In our study, awareness of HPV infection, HPV testing, and HPV vaccination increased significantly with advancing class level, consistent with findings reported among medical students in other settings^5,9,13^. These findings underscore that awareness—alongside academic progression—plays a critical role in shaping health-related knowledge among medical students.

In contrast, studies conducted among students from non-health-related departments have reported substantially lower HPV knowledge and awareness levels, generally ranging between 15 and 40%^10,18,19,20,21^. This highlights the importance of structured health education in fostering informed preventive attitudes and professional competence. The observed increase in HPV knowledge among senior students may be related to exposure during obstetrics and gynecology courses and clinical rotations, where students encounter HPV infection and cervical cancer cases more directly.

Our findings not only confirm the expected increase in knowledge levels with academic progression, but also demonstrate that prior awareness of HPV and HPV testing significantly enhances knowledge beyond the effect of advancing academic year. By explaining 41% of the variance in HPV knowledge, our model demonstrates substantial explanatory capacity and provides a meaningful contribution to the existing literature. Identifying the core determinants of HPV knowledge may facilitate their integration into medical curricula, thereby fostering early awareness and preventive competence among future physicians.

This study represents the first regional assessment of HPV knowledge among medical students. However, its single-center design and local context may limit external validity. Additionally, voluntary participation and the use of convenience sampling may have introduced selection bias.

## Data Availability

The datasets generated and/or analyzed during the current study are available from the corresponding author upon reasonable request.
